# Prioritization of deleterious mutations informs genomic prediction and increases the rate of gain in common bean (*Phaseolus vulgaris* L*.*), a simulation study

**DOI:** 10.1007/s00122-026-05329-z

**Published:** 2026-08-03

**Authors:** H. Cordoba-Novoa, V. Hoyos-Villegas

**Affiliations:** 1 Department of Plant Science, McGill University, Montreal, Canada; 2 Department of Plant, Soil and Microbial Sciences, Michigan State University, 1066 Bogue St, East Lansing, MI USA

## Abstract

**Key message:**

Predicted deleterious mutations (SNPs) have different distributions of effects compared to random SNPs based on population composition. Variant prioritization of markers based on deleterious scores can improve the prediction of yield. Favoring mating schemes between parents with fewer highly deleterious mutations can increase the rate of genetic gain.

**Abstract:**

The study of mutations is fundamental to understanding evolution, domestication, and genetics. Characterizing mutations has potential to accelerate breeding programs through selection and purging deleterious mutations (DelMut). We investigated how predicting DelMut in breeding populations informs genomic prediction (GP) increasing the rate of genetic gain. DelMut were annotated in three independent common bean populations using a previously developed random forest (RF) model for common bean incorporating phylogenetic and protein information. Deleterious scores from the RF model were around 0.25, with the top 1% (*highly* DelMut) of variants scoring between 0.78 and 0.82 among populations. All populations showed variation in the number of *highly* DelMut per line (max. 13–197) and in genetic load. We assessed the impact of incorporating a priori information on DelMut for variant prioritization and weighting in GP models for yield and flowering time. Stochastic simulations were conducted to evaluate how designing mating schemes based on variable numbers of DelMut per parent can affect genetic gain. Variants with higher predicted scores had significantly different effect distributions compared to random or lower-scored markers. Simulated breeding cycles showed that selecting parents with fewer *highly* DelMut consistently increases the rate of genetic gain, and depending on the population, can be superior to phenotypic selection. These results highlight the potential of DelMut information for variant prioritization and the optimization of common bean breeding programs. The approaches we developed can be applied to other species to improve the efficacy of crop improvement.

**Supplementary Information:**

The online version contains supplementary material available at 10.1007/s00122-026-05329-z.

## Introduction

The study of mutations plays a paramount role in understanding the evolution and adaptation of species, as well as genetic and phenotypic variation (Charlesworth et al. 1993; Gossmann et al. 2012; Piganeau and Eyre-Walker 2003). Depending on their effect, mutations can be neutral, advantageous, or deleterious. The distribution of the fitness effect (DFE) of mutations helps explain the proportion of mutations that can be beneficial or detrimental depending on environmental forces (Eyre-Walker and Keightley 2007; Krasovec et al. 2016). Nonsynonymous mutations (both advantageous or deleterious) are under strong selective pressures. Strongly advantageous mutations are usually rare (Eyre-Walker 2006), and due to their effect, highly deleterious mutations (DelMut) are purged depending on the plant mating systems, and population size and structure (Crow 1970). In self-pollinated plants, inbreeding may expose dominant deleterious mutations in the homozygous state, leading to purging by natural or artificial selection. Outcrossing species maintain high levels of heterozygosity that can maintain DelMut masked. However, mildly recessive DelMut with subtle effects can escape purifying selection and are accumulated, known as the genomic genetic load (Glémin 2003).

Multiple factors affect the accumulation of mildly DelMut. In crop species, domestication and selection bottlenecks contribute to the increase of inbreeding in populations, which in turn reduces the effective population size and effectiveness of purifying selection, leading to the accumulation of weakly DelMut (Charlesworth and Charlesworth 1999; Kono et al. 2016). The accumulation patterns and potential role of DelMut in phenotypic variation are topics of particular interest in crop improvement. Studies on the characterization of DelMut in diverse populations have been conducted in several species with varying accumulation patterns depending on the mating system and breeding methods (Dwivedi et al. 2023). In self-pollinated species such as common bean and soybean, breeding has reduced the number of DelMut, but mildly DelMut are fixed and accumulated (Cordoba-Novoa et al. 2025; Kim et al. 2021).

The accumulation of DelMut can limit selection and hinder the genetic gains in breeding programs (Moyers et al. 2018; Zhu et al. 2022). Domestication and improvement increase inbreeding and reduce the effective population size, which in turns reduces the effectiveness of natural selection and leads to the accumulation of mildly and slightly DelMut (Dussex et al. 2023; Renaut and Rieseberg 2015). Depending on the breeding purpose and the target environment and conditions, the effects of DelMut can be associated with their effect on the overall plant fitness. Fitness can be defined as the ability of an organism to survive and reproduce (Orr 2009). The level of fitness of an individual is related to the proportion of the next generation represented by its offspring and depends on the contextual framework. In a breeding context, for instance, plants able to survive biotic and abiotic stress or yield more in different environments are expected to have a higher fitness. That same ability to produce more can be affected by the genetic load of the individual or the population if analyzed as a whole (Dwivedi et al. 2023).

The identification and characterization of DelMut in crop species raises the question of how genetic gain in breeding programs can be further accelerated (Johnsson et al. 2019; Wallace et al. 2018). The targeted removal of DelMut using new genomic techniques (NGT) such as genome editing is one of the avenues for crop improvement (Gao 2021; Glaus et al. 2025; Johnsson et al. 2019). Other approaches aim to enhance the prediction ability (PA) of genomic selection (GS) models. Some studies have considered the use of predicted DelMut to subset markers for GS or their inclusion as fixed effects in the model (Valluru et al. 2019; Wu et al. 2023). Methods that modify the relative importance of markers in the model through the inclusion of posterior variances or the modification of genomic relationship matrices (GRM) have also been considered (Edwards et al. 2016; Long et al. 2023; Yang et al. 2017). The different approaches have shown varying levels of efficacy depending on the trait, the populations, and the environments.

In breeding programs, decisions and final results are simultaneously influenced by various parameters. Stochastic simulations have been adopted as tools to simulate breeding scenarios and probable outcomes for optimizing breeding programs (Covarrubias-Pazaran et al. 2022; Hassanpour et al. 2023). Such simulations have the advantages of recreating entire populations with genotypic and phenotypic data at the individual level, which provides precise predictions of the consequences of proposed changes at different stages, such as crossing, evaluation, and selection (Liu et al. 2019; Vieira et al. 2025). Stochastic simulations have been employed for the simulation and optimization of animal (Gorjanc et al. 2018; Johnsson et al. 2019), and plant breeding programs, including rice (Fritsche-Neto et al. 2024), sweet corn (Peixoto et al. 2024), soybean (Silva et al. 2021), and common bean (Chiaravallotti et al. 2024; Lin et al. 2023) considering the adoption of GP frameworks.

As a predominantly self-pollinating species, common bean may accumulate DelMut with varying effects. As mildly DelMut can accumulate in different genomic regions, the purging of DelMut from breeding populations and elite material has the potential to increase the effectiveness of breeding programs. However, no studies on the potential practical applications of the inclusion of DelMut in plant breeding pipelines have been conducted. Thus, we investigated the potential impact of using information derived from the prediction of DelMut for optimizing breeding programs. Our objective was to predict DelMut in different common bean breeding populations and evaluate how genomic prediction (GP) could benefit from variant prioritization in the model. Additionally, we hypothesize that selecting parents and designing mating schemes based on a priori information about the presence of DelMut could accelerate the rate of genetic gain over time in breeding programs.

## Materials and methods

### Phenotypes and genotypes

Three independent common bean breeding populations with publicly available phenotypic and genotypic data were used in this study. The populations have different genetic backgrounds, breeding histories, and genotypic datasets and have been evaluated in different environments. The black bean MAGIC population was developed at McGill University, Canada, with eight parents from the Middle American Diversity (MDP) panel (Moghaddam et al. 2016). Founders were selected to maximize allelic diversity and crossed in a multi-funnel scheme (half-diallel). A total of 18 families and 532 recombinant inbred lines (RIL) were advanced. Field experiments were conducted in Canada in 2024 (Cordoba-Novoa et al. 2025). For the present study, the evaluations from the Sainte Anne de Bellevue, QC (SAB) location were used based on data quality and the experimental design. Flowering was evaluated as the number of days from planting until 50% of the plot plants had at least 50% of their flowers open (DTF). Yield was recorded as kg/Ha based on the plot weight. Best Linear Unbiased Estimators (BLUEs) were calculated by fitting the following mixed-effect model in the *lme4* *R* package (Bates et al. 2015) and an alpha-lattice design. Means were corrected for further analysis.

$$Y_{ijk} = \, \mu \, + \, \tau_{i} + \, \gamma_{j} + \, \rho_{k(j)} + \, e_{ijk}$$

where *µ* is the overall mean, ${\tau}_{i}$ is the fixed effect of the *i-th* genotype, ${\gamma}_{j}$ is the random effect of the *j-th* replicate, *ρ*_*k(j)*_ is the random effect of the block within the replicate, and ${e}_{ijk}$ is the experimental error associated with observation *ijk.*

The MAGIC RILs were skim-sequenced using 150 paired-end PCR-free libraries in the Illumina platform and imputed using a Practical Haplotype Graph (PHG) built from Nanopore and Illumina reads from the eight founders (Bradbury et al. 2022; Cordoba-Novoa et al. 2025). A total of 1.5 million SNPs were available for the present study.

The second population was the Vivero Equipo Frijol (VEF). VEF is an elite Andean population that is part of the bean breeding program of the International Center of Tropical Agriculture (CIAT). Details on the population and phenotypic evaluation can be found in Keller et al. (2020). For the present study, we used the VEF phenotypic data for 346 genotypes evaluated in Darien (Valle del Cauca, Colombia) under the medium-phosphorous level (DAR16C_mdP location). This location was selected because it included the largest number of evaluated genotypes and had similar prediction abilities to other DAR16C evaluation environments. BLUE-corrected means for DTF and yield (kg/Ha) were available and directly used for our analysis. As previously reported, the VEF population was genotyped using genotype-by-sequencing (GBS) with the *ApeKI* restriction enzyme and Illumina sequencing. After quality control, 5,820 SNP markers were available.

The third population was the Cooperative Dry Bean Nursery (CDBN), a multi-environment trial (MET) grown for over 70 years in the USA and Canada. MacQueen et al. (2020) reported the data analysis strategies for four decades of the CDBN evaluation. For yield, we used phenotypic data from 318 genotypes evaluated at the MTSI (Sidney, Montana, USA) location, and for DTF, we used data from 226 genotypes evaluated at the WYPO (Powell, Wyoming, USA) location. These locations had the highest number of observations (datapoints) for each trait. Due to the unbalanced nature of the data, Best Linear Unbiased Predictors (BLUP) were calculated across years. MacQueen et al. (2020) genotyped the CDBN accessions by re-analyzing raw sequencing from previous reports and sequencing some genotypes with a dual-enzyme (*MseI* and *TaqI*) GBS approach. After variant calling and QC, a total of 1.2 million SNPs were available.

### Population structure and LD analyses

As mentioned above, the populations used were previously reported and characterized to a certain extent with independent genotypic datasets in each case. To facilitate the analysis of the results, we evaluated population structure using principal component analysis (PCA) implemented in TASSEL v5 (Bradbury et al. 2007) and pairwise linkage disequilibrium (LD) in GAPIT v3 (Wang and Zhang 2021). LD was plotted using the R package LDheatmap (Shin et al. 2006). For population structure and LD evaluation, a minimum distance of 10 kbp between SNPs was set to reduce datasets to a similar number of equally spaced markers while balancing marker number and density, and maintaining the LD patterns.

### Deleterious mutations (DelMut) prediction and genetic load

To identify putatively DelMut, we used two Random Forest (RF) models reported by Cordoba-Novoa et al. (2025). The models are based on conservation constraint, one trained based on the Middle American and another on the Andean Common bean genome reference. The Middle American RF model was used for the MAGIC population, whereas the Andean RF model was used for the VEF and the CDBN. The model choice depends on the genetic background and the reference genome (UI111–Middle American or G19833–Andean) used for variant calling in each population. Briefly, for the model implementation, Sorting Intolerant From Tolerant For Genomes (SIFT4G; Ng and Henikoff 2003) scores and unified representation (UniRep) embeddings (Alley et al. 2019) are obtained for each genotypic dataset and later loaded in the RF models to calculate a deleterious score. As described by Cordoba-Novoa et al. (2025), the model predicts scores from 0–1 for mutations in coding regions and one of the advantages of the model is to provide a continuous range of deleteriousness rather than a binary classification of mutations. This allows more flexibility in the characterization of putatively deleterious mutations, where thresholds can be defined based on multiple factors and the researcher’s criteria. The higher the value from zero to one, the more deleterious an allelic change is predicted to be at an evolutionary constraint site. Despite having a continuous range of deleterious scores and to prioritize some putatively deleterious mutations, the SNPs in the top 1% of the deleterious were classified as *highly* deleterious (*highly* DelMut–HDelM) based on the specific and independent distribution of the scores in each population and not default parameters.

To obtain a genome-wide estimation of the deleterious burden in each population, we estimated the homozygous (Hom), heterozygous (Het), and total genetic load per genotype (entry) by adding all the predicted scores according to the allelic state (Hom or Het) in each line. Following a similar approach as Wu et al. (2023), if the alternative allele is not present (or is the reference), the score is not added to the load calculation. Despite common bean being a predominantly selfing species, some low outcrossing rates may occur, and some heterozygous sites can be captured in high-density sequencing. In this case, heterozygous alleles were considered to have a contribution of 50% (0.5 factor in the total genetic load) following a standard additive model.

$${\mathrm{Hom}} \cdot {\mathrm{GenLoad}} = \sum\limits_{{i = 1}}^{{i = n}} {{\mathrm{Del}} \cdot {\mathrm{Score}}_{i} } ,{\mathrm{when}}\;{\mathrm{genotype}}\;i = {\mathrm{Homozygous}}\;{\mathrm{Alternative}}$$

$${\mathrm{Het}} \cdot {\mathrm{GenLoad}} = \sum\limits_{{i = 1}}^{{i = n}} {{\mathrm{Del}} \cdot {\mathrm{Score}}_{i} } {\mathrm{when}}\;{\mathrm{genotype}}\;i = {\mathrm{Hterozygous}}\;{\mathrm{Alternative}}\;\{ 1\}$$

$$\mathrm{T}\mathrm{o}\mathrm{t}\mathrm{a}\mathrm{l} \mathrm{G}\mathrm{e}\mathrm{n}\mathrm{L}\mathrm{o}\mathrm{a}\mathrm{d}=\mathrm{H}\mathrm{o}\mathrm{m}\cdot \mathrm{G}\mathrm{e}\mathrm{n}\mathrm{L}\mathrm{o}\mathrm{a}\mathrm{d}+0.5 x \mathrm{H}\mathrm{e}\mathrm{t}\cdot \mathrm{G}\mathrm{e}\mathrm{n}\mathrm{L}\mathrm{o}\mathrm{a}\mathrm{d}$$

where $\mathrm{H}\mathrm{o}\mathrm{m}\cdot \mathrm{G}\mathrm{e}\mathrm{n}\mathrm{L}\mathrm{o}\mathrm{a}\mathrm{d}$ is the homozygous genetic load, $\mathrm{H}\mathrm{e}\mathrm{t}\cdot \mathrm{G}\mathrm{e}\mathrm{n}\mathrm{L}\mathrm{o}\mathrm{a}\mathrm{d}$ is the heterozygous genetic load, $\mathrm{D}\mathrm{e}\mathrm{l}\cdot \mathrm{S}\mathrm{c}\mathrm{o}\mathrm{r}\mathrm{e}$ is the deleterious score calculated with the RF model, homozygous alternative is a site with homozygous state for the alternative allele, and heterozygous alternative is a site with heterozygous state for the alternative allele.

Due to population structure in CDBN, differences in the mean genetic load between subpopulations were compared accounting for relatedness. For this, the following mixed linear model was fit using rrBLUP with and without the inclusion of cluster (from PCA) as a fixed effect and the Genomic Relationship Matrix (GRM) calculated from the genotypic data (see below). The models (with and without clusters as fixed effects) were compared with a Likelihood Ratio Test (LRT) to determine the significance in the total genetic load differences.

y=Xb+u+e

$$u \sim N(0,{\sigma}_{g}^{2}K)$$

e∼(0,I)

$$\mathrm{V}\mathrm{a}\mathrm{r}\left(y\right)={\sigma}_{g}^{2}K+{\sigma}_{e}^{2}I$$

where *y* is the vector of the total genetic load in each individual, *X* is he fixed-effect matrix (cluster for mean comparison), *b* is the fixed-effect coefficients, *u* is the random genetic effect, *K* is the GRM, and *e* is the residual error.

### Effect size estimation and tests

Following the approach proposed by Valluru et al. (2019) the effect size of the SNPs was estimated from the relationship between phenotypes and genetic markers using a RR-BLUP model in the R package rrBLUP v4.6 (Endelman 2011).

y=μ+Zu+e

where *y* is the vector of BLUEs or BLUPs for yield or DTF, μ is an intercept vector, *Z* is an *n* × *p* incidence matrix with genotypes coded as {− 1,0,1} under an additive model, and *u* ~ *N*(0, *I* ${\sigma}_{u}^{2}$) is the vector of marker effects where ${\sigma}_{u}^{2}$ is the variance of the common distribution of marker effects estimated using restricted maximum likelihood (REML).

For each population, the distributions of the effects of SNPs with and without score were compared. Since the number of SNPs with scores is lower, random subsets of equal size of SNPs without scores were used (subsampling). The minor allele frequency (MAF) was checked among subsets to verify it was near equal to avoid bias. SNPs scored for DelMuts were further subset, and effects re-calculated to compare the effect distributions between the SNPs with the top 30% DelMut score and the remaining 70% based on weight distribution and percentiles. Differences between distributions were evaluated using the Kolmogorov–Smirnov (KS) test in *R*.

### Genomic relationship matrix dimension reduction prior to genomic prediction

For the VEF population, no further filtering or reduction was applied to the SNP dataset. Based on the original GP study for VEF that showed that the marker number (higher than 1K markers) did not affect the model PA, and to give enough room to accommodate SNPs with deleterious scores, subsets of 3,000 SNPs were used for the iterations (rounds of subsampling, Keller et al. 2020). Due to the high number of SNPs and to alleviate computational burden in prediction, the MAGIC and CDBN genotypic datasets were reduced based on LD using the *-indep-pairwise* tool in Plink v1.9 (Chang et al. 2015) with a window size of 50 kb, step of 1, and an *r*^2^ threshold of 0.3, similar to previous reports for common bean (Keller et al. 2020). When using the RF model for the prediction of DelMut, not all the SNPs will have an assigned deleterious score due to their genomic location and evolutionary constraint. Given this and that one of our main goals is to evaluate the potential benefits of the prediction of DelMut, after reducing the MAGIC and CDBN marker datasets, SNPs with scores that filtered out were re-incorporated into the baseline dataset. In each iteration of these two populations, we used subsets of 7,000 randomly selected markers, maintaining an equal number of SNPs with and without deleterious scores (weighted and unweighted, respectively).

### Genomic prediction

**Fig. 1:**
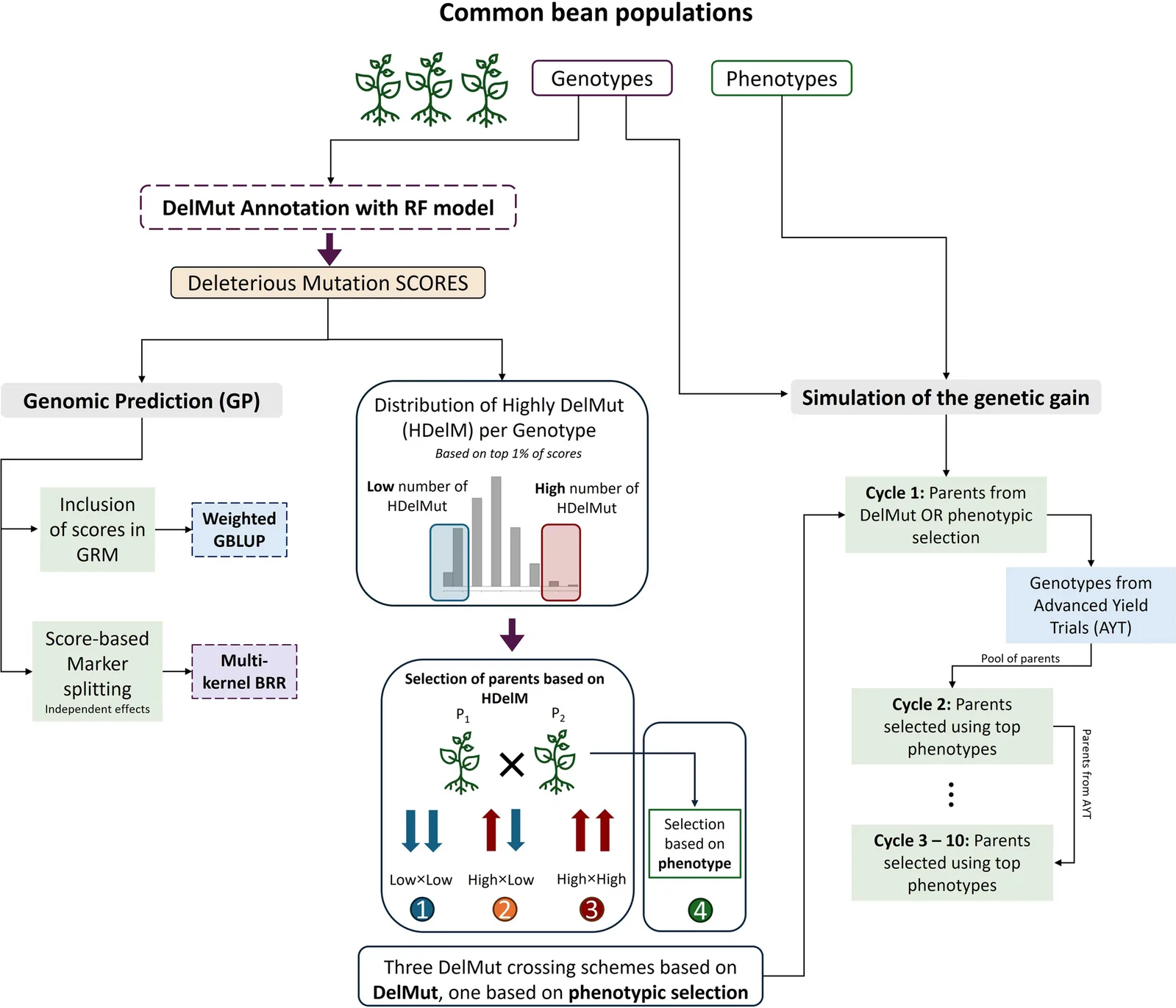
Overview of the implemented methods for the prediction of deleterious mutations in three common bean populations and its application in genomic prediction and simulations

To evaluate the potential of incorporating information from DelMut into genomic prediction (GP), a Genomic Best Linear Unbiased Prediction (GBLUP) and a Bayesian Ridge Regression (BRR, also known as Gaussian prior) model were used (Fig. 1). GBLUP and BRR can be equivalent models under certain circumstances, BRR being the Bayesian version of RR-BLUP, which in turn is equivalent to GBLUP. However, the methods were chosen based on how each one can model markers based on the DelMut predicted scores (direct marker matrix or GRM) as described below. The following GBLUP model was implemented in ASReml (Butler et al. 2023).

y=Xb+Zu+e

where *y* is the vector of phenotypic BLUEs or BLUPs, *X* is a design matrix relating the fixed effects to each genotype, *b* is the vector of fixed effects, *Z* is a design allocating the records of genetic values, *u* is the vector of additive genetic effects for a genotype, and *e* is the vector of normally distributed residuals. In the model, $\mathrm{v}\mathrm{a}\mathrm{r}\left(u\right)=G{\sigma}_{\mathrm{g}}^{2}$ where *G* is the genomic relationship matrix (GRM) and ${\sigma}_{\mathrm{g}}^{2}$ is the genetic variance for the model. The GRM was constructed using the additive method from VanRaden (2008) in the R package *AGHmatrix* (Amadeu et al. 2023) as follows:

$$G= \frac{MDM{\prime}}{\sum 2{p}_{i}{q}_{i}}$$

where *M* is the centered genotype matrix coded as 0, 1, and 2 for the homozygous reference allele, heterozygous, and homozygous alternative allele, respectively. *D* is the identity matrix, and *p* and *q* are the allele frequencies.

The following BRR model was implemented in the BGLR package (Pérez and De Los Campos 2014).

y=Xw+e

where *X* is the design matrix, and *e* is the independent and identical distributed Gaussian error. *w* is the vector of marker effects (model parameters) where $w \sim N(0, {\lambda }^{-1}I$). In the model, *y* is the vector of phenotypic values with ${y}_{i}\sim N\left({x}_{i}^{\top }\mathrm{w}, {\sigma }^{2}\right)$. The number of Markov Chain Monte–Carlo iterations per model was 100,000 with the first 5,000 as burn-in and a thin parameter of 50. A linear BRR regression (ETA parameter) was applied on the marker incidence matrix. Additional parameters were kept as default.

To evaluate models performance, predictions were run with ten iterations with a tenfold cross-validation (CV). In each iteration, SNP dataset was sampled and used for training according to the model and approach. For the CV, in VEF and MAGIC populations, the population was randomly split into 70% and 30% for the training and testing datasets. Due to the high population structure and stratification in CDBN, and to limit within-cluster leakage, we trained within randomly sampled 70% subsets from non-focal clusters and predicted the 30% held-out genotypes from the left-out clusters. This controls for potential bias in prediction ability (PA) coming from samples within the same subpopulation (cluster) as the model is trained in individuals from a single cluster, but tested on individuals from a different cluster. The PA was determined in each fold by Pearson’s correlation between the predicted and the observed phenotypic values across iterations, and the mean PA across folds was reported.

### Inclusion of deleterious mutations information in genomic prediction

Based on the model, two strategies for the inclusion of DelMut information were implemented (Fig. 1). For the GBLUP model, new GRMs were constructed weighing the SNPs based on their predicted DelMut scores from the RF model according to (Liu et al. 2020) for the VanRaden method used using the following formula:

$${G}_{\mathrm{W}}= \frac{M{D}^{*}M{\prime}}{\sum 2{p}_{i}{q}_{i}}$$

where ${G}_{\mathrm{W}}$ is the weighted *G*-matrix and ${D}^{*}=DT$, where *T* is a diagonal matrix with diagonal element *j* being the predicted DelMut score (weight) for each SNP. To avoid eliminating markers with no score and lose information that could affect prediction performance and comparisons to unweighted models, a baseline value of 0.001 was added to all markers. In this way, all markers are considered in the GRM, but the markers with a score are differentially represented based on the DelMut score. The weights were normalized to have mean 1, so that the overall ${G}_{\mathrm{W}}$ remains close to that of the standard VanRaden method. Here weights are only included in the numerator, allowing SNPs with higher scores to contribute more to genomic covariance, while keeping variance components comparable to those obtained with an unweighted *G* (Liu et al. 2020). For the weighted GBLUP model, additional non-linear score transformations (rank-based and exponential) and inclusion of the calculated effect of each marker were explored. Due to the lack of effect or improvement in the model PA, results were not included.

For the second strategy, instead of directly including the scores in the model, scores were used to guide marker partitioning in the BRR model. Unlike GBLUP, the BRR model starts from the genotype matrix rather than a GRM. To inform the model about DelMut, the marker dataset was split into scored and unscored SNPs and defined as two different random effects in a two-level list in the model, following a multi-kernel approach (Pérez and De Los Campos 2014) according to the equation below. Here, two different incidence matrices (markers with and without score) are included in the model (ETA parameter in R) and a BRR linear regression is applied to each one, allowing independent modeling based on the model below. To avoid potential size bias, in each iteration, the SNP subsets were randomly sampled and kept to the same number of markers.

$$y=\mu +{Z}_{\mathrm{s}}{\beta}_{\mathrm{s}}+{Z}_{\mathrm{u}}{\beta}_{\mathrm{u}}+\varepsilon$$

where *y* is the vector of phenotypic BLUEs or BLUPs, ${Z}_{\mathrm{s}}$ and ${Z}_{\mathrm{u}}$ are the *n* × *p* incidence matrix for scored and unscored SNPs, ${\beta}_{\mathrm{s}}$ and ${\beta}_{\mathrm{u}}$ are the vector of scored marker effects for scored and unscored SNPs, and *ε* is the vector of residuals with

$${\beta}_{\mathrm{s}} | {\sigma}_{{\beta}_{\mathrm{s}}}^{2}\sim \prod_{j=1}^{{P}_{s}}DE(0,\frac{{\sigma}_{{\beta}_{\mathrm{s}}}^{2}}{2})$$

and

$${\beta}_{\mathrm{u}} | {\sigma}_{{\beta}_{\mathrm{u}}}^{2}\sim \prod_{j=1}^{{P}_{u}}DE(0,\frac{{\sigma}_{{\beta}_{\mathrm{u}}}^{2}}{2})$$

### Simulation of the genetic gain

We evaluated how incorporating information on DelMut could improve selection and increase the rate of genetic gain over time. As proof of concept, we simulated a hypothetical basic breeding scenario using a pedigree breeding strategy with artificial selection for a quantitative trait in each generation (represented in Lin et al. 2023). Figure 1 depicts the overview of the simulations and crossing schemes. For the simulations, the initial crosses of the first breeding cycle are carried out between parents solely selected based on the number of *highly* DelMut (HDelM). Three independent crossing schemes (closed programs) based on number of HDelM were compared. First, genotypes with a high number of *HDelM* were crossed between them High × High (H × H); second, parents with a low number of *HDelM* were crossed Low × Low (L × L); and third, parents with a high number of *HDelM* were crossed with parents with a low number of *HDelM* High × Low (H × L).

Within each proposed scenario, the top (higher number of DelMut) and the bottom (lower number of DelMut) 20 parents were used for 20 crosses randomly assigned. The number of parents was chosen based on the founder set size of small-to-medium-sized breeding programs and distribution of the number of *HDelM*. Since we simulated a closed system and new parents were not introduced after the first cycle, no further selection based on DelMut content was made. From the second cycle, parents were selected from the advanced lines of the previous cycle based on the phenotype (top simulated parents are included in the next cycle). To compare the selection based on *HDelM* with a phenotypic selection strategy (based on BLUEs or BLUPs), an additional scheme of crosses between parents selected based on phenotype was also simulated. As before, in this fourth scenario, the top 20 parents with the highest yield or the lowest DTF (desired breeding outcomes) were randomly crossed (20 crosses) in the first breeding cycle, and subsequent selections were done based on phenotype as explained above for the three other scenarios.

In the simulations, 10 breeding cycles from initial crosses to advance yield trials (AYT) were simulated with 50 iterations in AlphaSimR (Chris Gaynor et al. 2021). The initial crosses were done with a progeny of 50 for an initial population size of 1,000. Following the pedigree breeding strategy mentioned before, 40% of the population was selected within families in F2 and 30% in F3. In F4, 25 families were selected, and 10 in the preliminary yield trials (PYT). Finally, a single family was selected in the Advanced Yield Trials (AYT) to develop a variety. The traits (Yield and Days to flowering) were modeled using an additive model. Since the objective of our simulations was to prove a concept and provide an initial baseline for the potential use of the annotation of deleterious mutations in breeding programs, no additional genetic models, re-introduction of germplasm, or inbreeding was considered. Real (not simulated) genotypic and phenotypic data collected for each population (VEF, MAGIC, CDBN) were used for the simulations. An initial population is created specifying the genetic map and the haplotypes from the genotypic data of each population. AlphaSimR will simulate normally distributed phenotypes based on a provided mean. Here, we used the observed mean from the BLUES in each population and replaced the individual simulated values by the observed BLUEs/BLUPs in the ‘pheno’ element. Other environmental and residual structure parameters were kept as default.

Average genetic gain was calculated per cycle as the difference between the mean genetic value of the varieties and the mean genetic value of the parents in each cycle. Cumulative genetic gain was calculated in each cycle by adding the previous cycle’s shift in genotypic value. The trait heritability is experiment-dependent, and it is defined for a specific population, set of environments, and experimental design. To keep some comparisons stable among populations, a main broad-sense heritability (H^2^) was set as 0.25 for yield and 0.45 for flowering based on previous reports for common bean (Delfini et al. 2021; Kamfwa et al. 2015; Keller et al. 2020; Raggi et al. 2019). However, we also evaluated simulations using H^2^ values of ± 0.1 in each trait. The genetic map reported by Diaz et al. (2020) was used for simulations.

## Results

### Populations used for predictions and simulations vary in structure, LD, and phenotypes

**Fig. 2:**
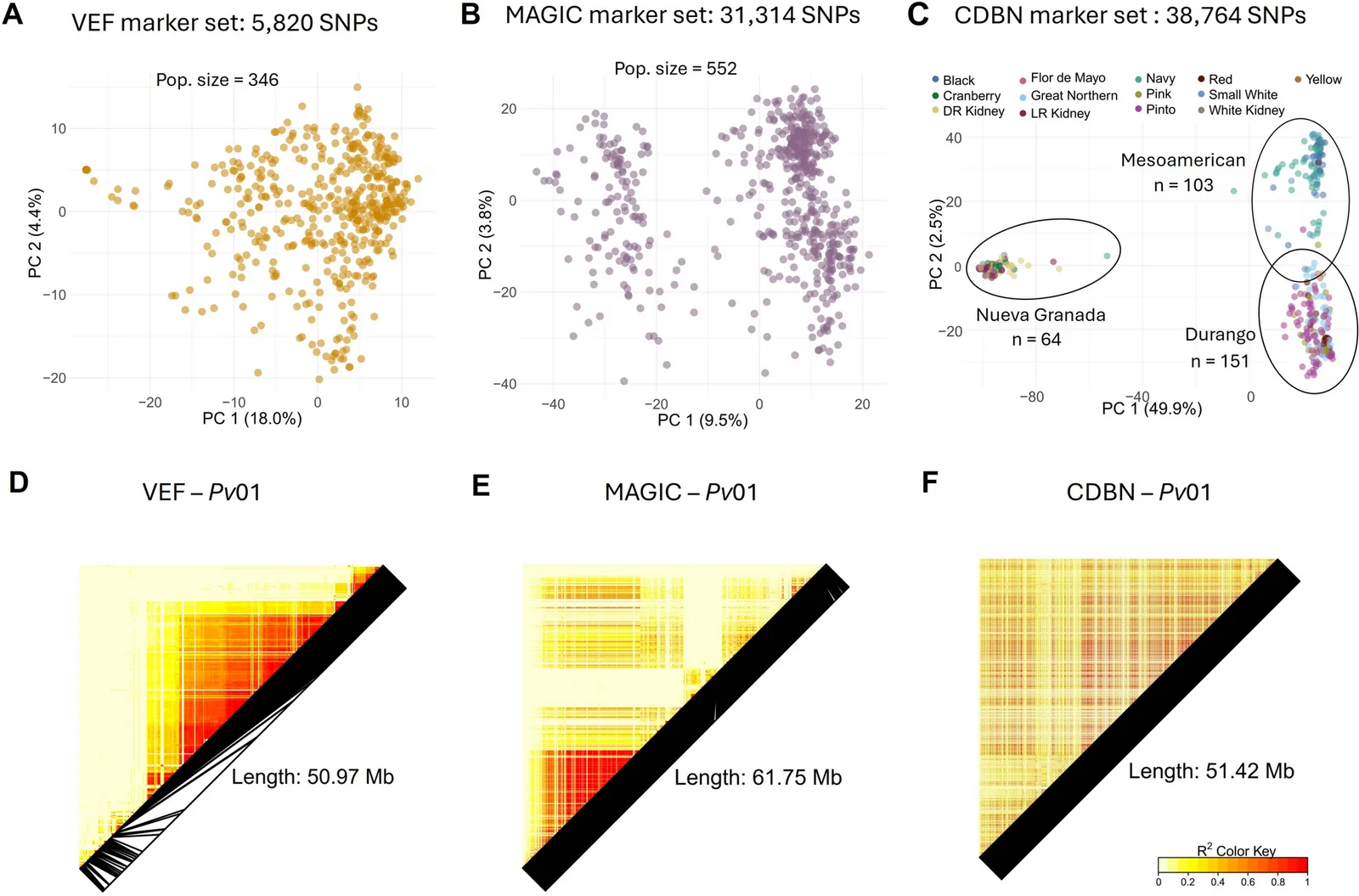
Principal component analysis (PCA) for population structure in Vivero Equipo Frijol (VEF; **A**), Black bean MAGIC population (**B**), and Cooperative Dry Bean Nursery with defined market classes (CDBN; **C**). Linkage disequilibrium (LD) heatmaps for chromosome 01 (Pv01) in VEF (**D**), MAGIC (**E**), and CDBN (**F**). The SNP dataset is independent for each population

The three populations, VEF, MAGIC, and CDBN, have different breeding histories, genetic backgrounds and were genotyped using various methods, which makes direct comparisons challenging. However, different population structures and LD patterns were observed within each population. In the PCA, VEF had a main single cluster with a few lines on the far left and explained variation of 22.4% between PC1 and PC2 (Fig. 2A). The MAGIC population had two weak subpopulations, mainly based on the PC1, that explained only 9.5% of the variation (Fig. 2B). The CDBN had two main subpopulations based on PC1 (49.9%) where one of them was slightly split based on PC2 (2.5%), for a total of 52.4% of the variation in both PCs (Fig. 2C).

Despite differences in marker density, similar chromosome lengths were observed in the LD analysis of each population. Different LD patterns were identified in each population (Figs. S1-S3). Figure 2 D-F shows the LD heatmap for *Pv*01 in each population as an example of the varying patterns observed in each population. For instance, a big cluster of markers in high LD (*r*^2^ > 0.8) was observed in the middle of Pv01 for VEF, while a smaller cluster, still in high LD, was identified at the beginning of the chromosome for MAGIC. In CDBN, no clear and large LD blocks were observed.

Descriptions of the phenotypic data can be found in the original reports for each population (Cordoba-Novoa, 2025; Keller et al. 2020; MacQueen et al. 2020). Briefly, in VEF, yield ranged from 457.5 to 1,815 kg/Ha with a mean of 1,072.6 kg/Ha (CV = 22.9%). Flowering ranged between 35.4 and 40.2 days and a mean of 38.2 days (CV = 2.5%). In the MAGIC RILs, yield ranged between 544.7 and 3,177.5 kg/Ha, with a mean of 1,886.2 kg/Ha (CV = 20.1%), and DTF from 41.5 to 50.5 days and a mean of 45.7 days (CV = 3.9%). In the CDBN dataset, yield in the MTSI location had higher values compared to VEF and MAGIC, ranging from 2,063.3 to 3,832.8 kg/Ha and a mean of 3,065 kg/Ha (CV = 9.9%). In the WYPO location, DTF varied from 54.3 to 62.2 days, with a mean of 58.4 days (CV = 2.3%; Table S1).

### The prediction and distribution of deleterious scores is population dependent

**Fig. 3:**
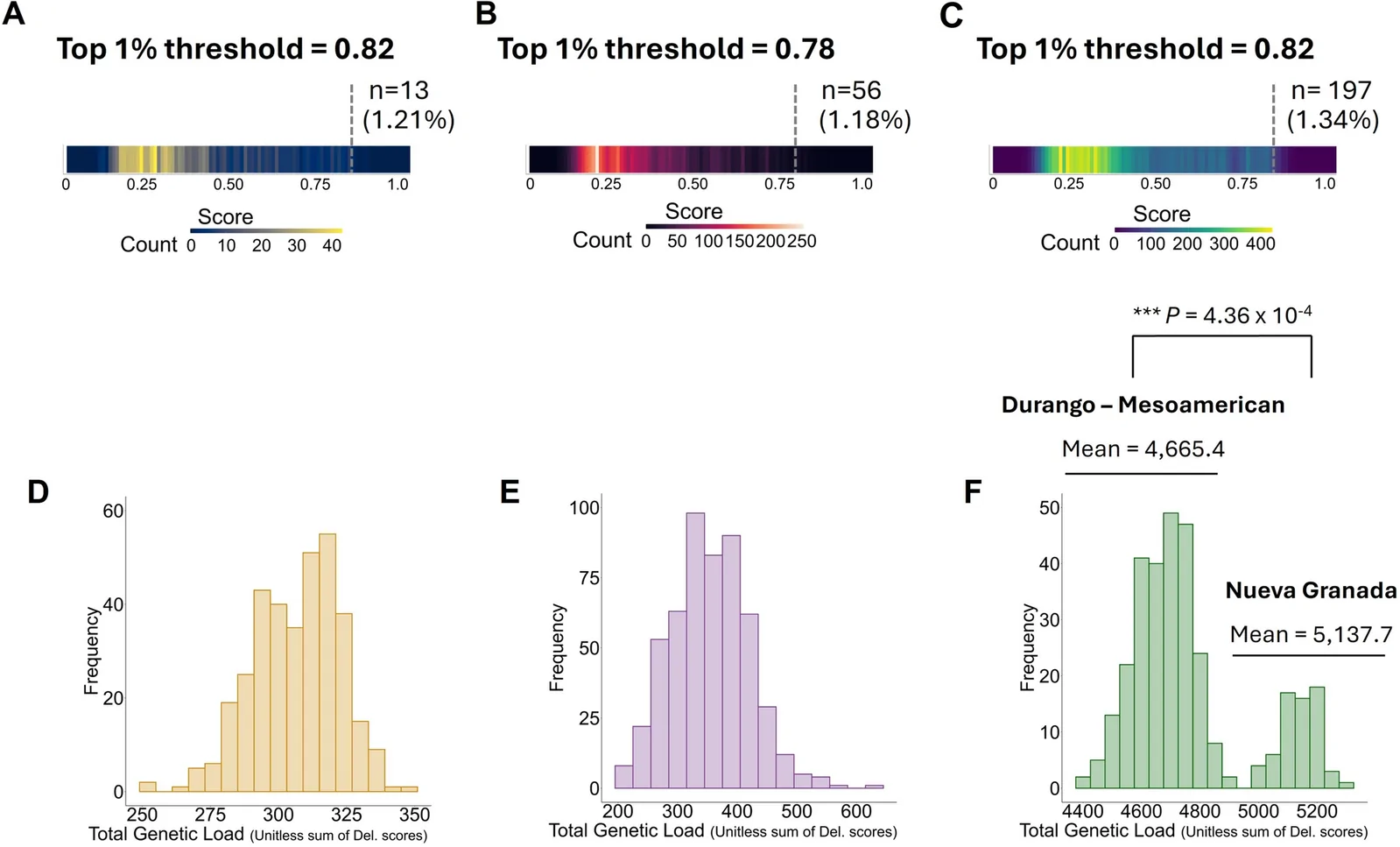
Prediction of deleterious mutations in three common bean breeding populations, Vivero Equipo Frijol (VEF; **A**, **D**), Black bean MAGIC population (**B**, **E**), and Cooperative Dry Bean Nursery (CDBN; **C**, **F**). Distribution of deleterious scores (**A**-**C**) and distribution of total genetic load (**D**-**F**). The values indicated in (**A**-**C**) (0.78–0.82) are the thresholds defined within each population to classify mutations as *highly* deleterious based on the 1% of the deleterious scores. The SNP dataset is independent for each population

The number of annotated SNPs with deleterious scores varied in each population depending on data availability. For the VEF population, 1,072 SNPs had a predicted score, 4,753 in the MAGIC population, and 14,740 in the CDBN dataset. Scores varied from zero to one, with most of the values around 0.25 in all three populations (Fig. 3A-C). Due to a higher marker density, in the CDBN there was a wider score distribution. Based on the top 1%, the threshold for *HDelM* was 0.82 for VEF and CDBN and 0.78 for MAGIC. In the three datasets, the number of *HDelM* was between 1.18 and 1.34% of the SNPs with a predicted score. For the total genetic load, the three populations showed normal distributions with values between 250 and 350 for VEF, 200 and 600 for MAGIC, and 4,400 and 5,200 for CDBN (Fig. 3D-F; Tables S2–S4). In the CDBN, only a few Middle American lines (*n* = 15) had total loads between 4,800 and 5,000, producing a bimodal distribution with mean genetic load of 4,665.4 and 5,137.7 in each peak (Fig. 3F; Table S4). The two peaks in the distribution are due to the two subpopulations identified based on PC1 from the PCA (Fig. 2C) where the cluster on the far left of the PCA plot had higher total genetic loads (> 5,000) and belonged mostly to race Nueva Granada (Fig. 3F; Table S4). Based on the relatedness-aware comparison, the mean total genetic load was significantly different between the two peaks. The homozygous genetic load calculated only with the *HDelM* showed the same distribution patterns for the three populations (Fig. S4).

Scored SNPs accumulated in 800 genes for the VEF population (Table S5), 7,639 for MAGIC (Table S6), and 4,943 in CDBN (Table S7). *HDelM* accumulated in 12 genes in VEF, 37 genes in MAGIC, and 143 genes in CDBN with 1–3 mutations per gene (Table S8).

### Distribution of SNP effects differs between scored and unscored variants

**Fig. 4:**
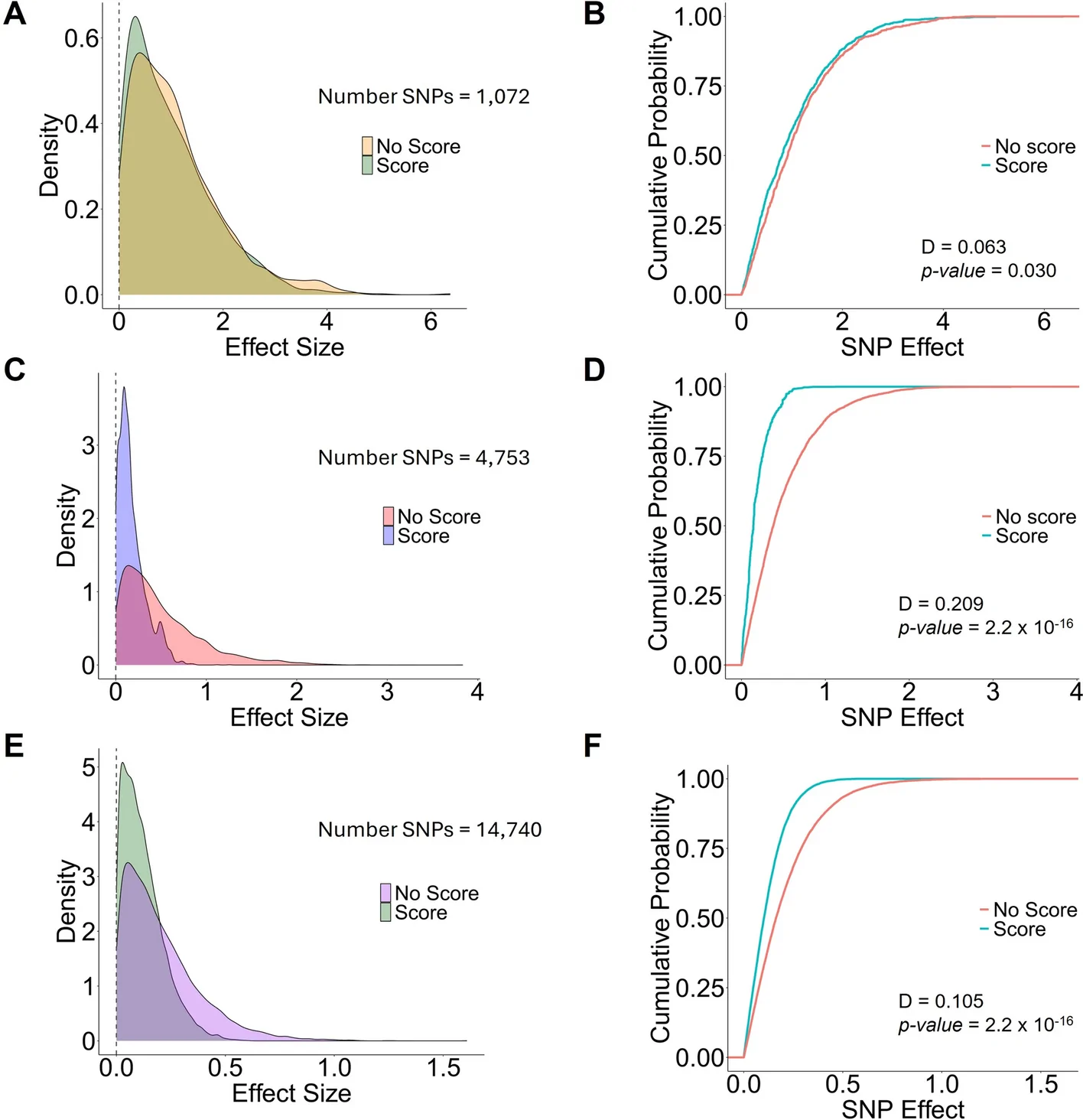
Distribution of the *absolute value* of the estimated effects of scored and unscored SNPs for yield in Vivero Equipo Frijol (VEF; **A**), Black bean MAGIC population (**C**), and Cooperative Dry Bean Nursery (CDBN; **E**). Cumulative probability for the distribution of the effects (**B**-**F**). *D* and *p* value correspond to the Kolmogorov–Smirnov (KS) test for distributions. The SNP dataset is independent for each population

**Fig. 5:**
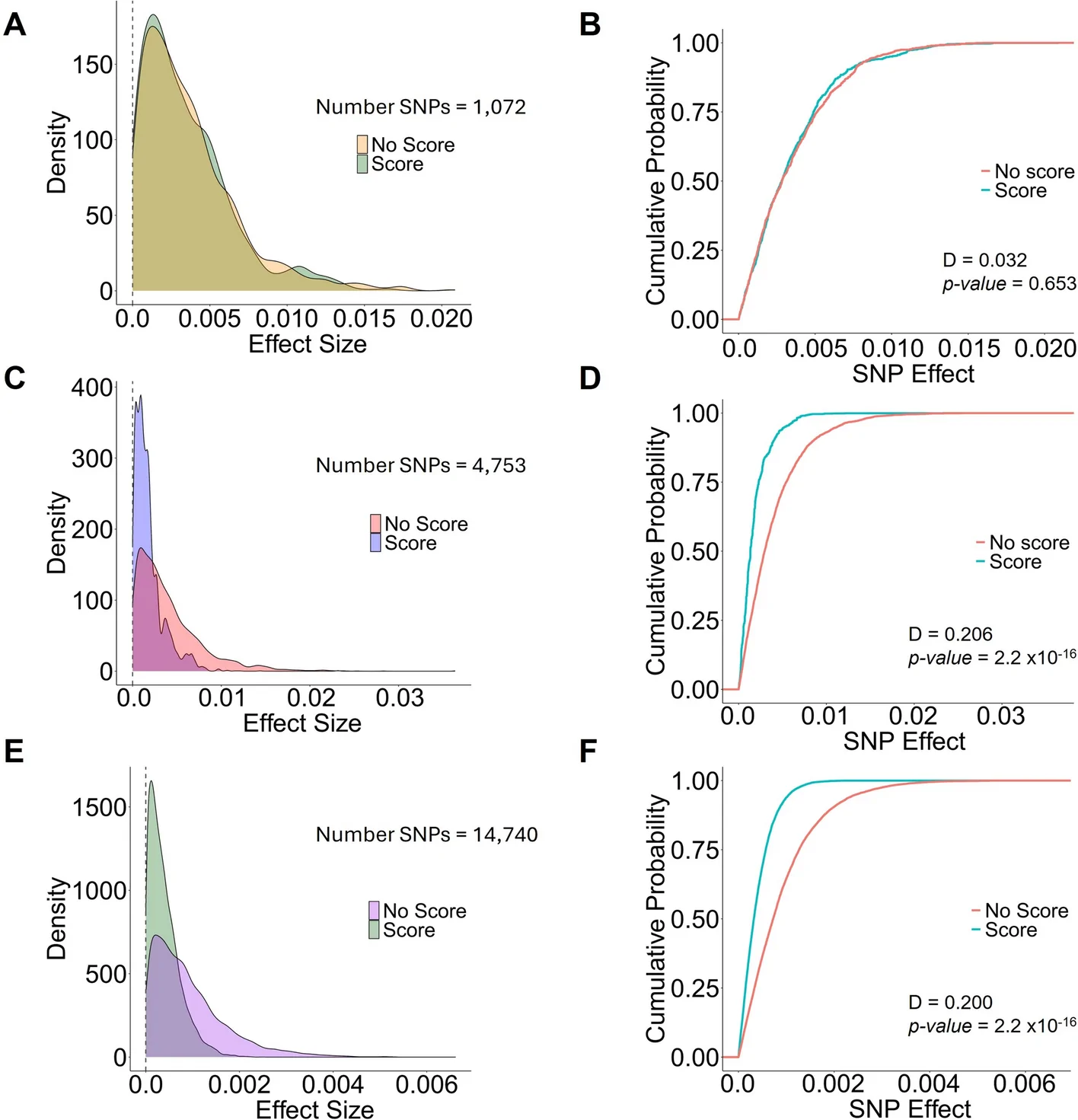
Distribution of the *absolute value* of the estimated effects of scored and unscored SNPs for days to flowering (DTF) in Vivero Equipo Frijol (VEF; **A**), Black bean MAGIC population (**C**), and Cooperative Dry Bean Nursery (CDBN; **E**). Cumulative probability for the distribution of the effects (**B**, **D**, **F**). *D* and *p* value correspond to the Kolmogorov–Smirnov (KS) test for distributions. The SNP dataset is independent for each population

The distributions of the estimated effects for scored and unscored SNPs followed normal distributions (Figs. S5–S8) with similar relationships with MAF between scored and unscored SNPs (Figs. S9–S10). To facilitate the analysis of the effects as magnitudes, the absolute values were considered. The absolute values of SNP effects showed differences between scored and unscored SNPs for yield and DTF. For yield, the effects of unscored SNPs were more evenly distributed compared to the scored SNPs. The effects for scored SNPs were mainly concentrated around zero, with a highly leptokurtic distribution and higher peaks (Fig. 4). Significant differences (*p* < 0.001) between the distributions were observed in the MAGIC and CBDN populations, with marginal differences in VEF (*p* < 0.05). When the scored SNPs were split based on the 30th percentile score threshold (0.39 for VEF, 0.38 for MAGIC, 0.5 for CDBN), a similar pattern was observed (Fig. S11. Markers with a higher score (predicted to be more deleterious) had a near zero peak compared to SNPs with lower score values. For DTF, the same behavior for the distribution of scored and unscored SNPs was observed in the three populations (Fig. 5). When comparing scored SNPs for the CDBN dataset, opposite to the trend, SNPs with scores < 0.5 had lower effects with a higher peak compared to SNPs with scores > 0.5 (Fig. S12-F).

### Genomic prediction models and traits differently benefit from the inclusion of deleterious mutations information

We investigated how incorporating previous information on DelMut could improve the prediction ability of genomic prediction models using two strategies: weighted GRM (GBLUP) and independent modeling of scored and unscored SNPs (BRR). For the BRR model, trace plots showed proper model convergence (Figs. S13, S14). The re-incorporation of scored SNPs that were filtered out during data reduction slightly increased the density of markers with MAF between 0.1 and 0.3 in MAGIC and 0.2 and 0.4 in CDBN compared to the originally filtered dataset (Fig. S17). This subtle change in density increased the mean MAF from 0.2207 to 0.2215 in MAGIC and from 0.1736 to 0.1769 in CDBN.

**Fig. 6:**
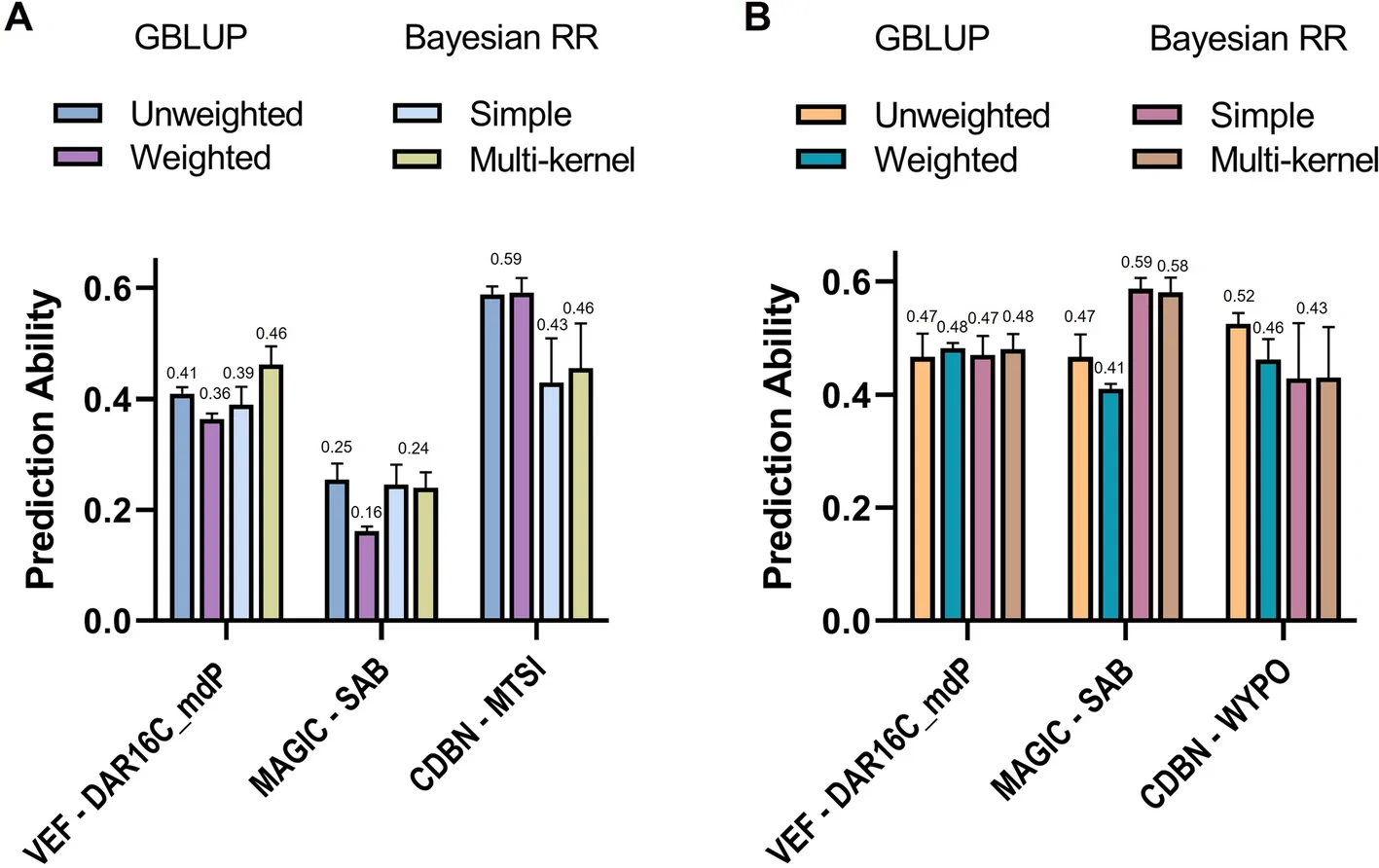
Prediction ability for yield (**A**) and days to flowering (**B**) using weighted and unweighted GBLUP, and simple and multi-kernel BRR models for the Vivero Equipo Frijol (VEF), Black bean MAGIC, and Cooperative Dry Bean Nursery (CDBN) populations. Vertical bars correspond to standard error

For yield, the unweighted GBLUP and simple BRR had similar PA for VEF (0.39–0.41) and MAGIC (0.24–0.25). In CDBN, the unweighted GBLUP had a higher PA (0.59) compared to the simple BRR (0.43). When incorporating the information from DelMut (scores as weights in the GRM of GBLUP, and multi-kernel partition based on scored and unscored SNPs for BRR), the PA changed depending on the model and the population. The inclusion of scores in the weighted GBLUP decreased the PA in 0.046 for VEF (0.36) and 0.093 for MAGIC (0.16) compared to the unweighted model (− 11.3% and -36.6%, respectively). However, in CDBN, the weighted GBLUP had a PA 0.0038 higher (+ 0.65% relative). The multi-kernel BRR model increased PA by 0.072 (+ 18.6% relative) in VEF (0.46) and by 0.026 (+ 6.0% relative) in CDBN (0.46) compared to the simple BRR. Similar to the weighted GBLUP, the multi-kernel BRR had a slight reduction in PA of 0.0057 (− 2.3% relative) in MAGIC. Overall, a multi-kernel BRR model had the highest PA for yield in VEF, the GBLUP for MAGIC, and a weighted GBLUP for CDBN (Fig. 6A).

For flowering (DTF), the unweighted GBLUP had PA of 0.47 for VEF and MAGIC and 0.52 for CDBN. Similar PAs were obtained for VEF using the simple BRR model (0.47), but a higher PA (0.59) was obtained for MAGIC and a lower PA for CDBN (0.43). The weighted GBLUP increased PA by 0.015 (+ 3.3% relative) in VEF (0.48) and decreased PA by 0.06 in MAGIC (0.41; − 12.0% relative) and in CDBN (0.46; − 11.9% relative). The multi-kernel BRR had marginal PA increases in PA for VEF (0.012 increase; + 2.27% relative) and CDBN (0.0013 increase; + 0.3% relative) and slightly decreased the PA for DTF in the MAGIC population by 0.006 (− 1.1% relative; Fig. 6B). In weighted GBLUP, restricting the analyses to scores above different thresholds did not improve results for yield or DTF (data not shown).

### The characterization of deleterious mutations potentially improves the rate of genetic gain over time

Genotypes from each population were selected solely based on the number of *HDelM* alleles as potential parents to simulate new *closed* breeding cycles with a pedigree method. This selection strategy with three scenarios (H × H, H × L, L × L) was also compared to a phenotypic selection based on the top desired phenotypes. In the VEF population, parents with a “Low” number had seven to eight mutations and “High” had 12–13 (Table S9). In the MAGIC population, selected parents in the “Low” group had 0–3 mutations, and those in the “High” group had 16–19 (Table S10). From the CDBN population, “Low” DelMut genotypes had 16–19 mutations, and *“*High*”* genotypes had between 31 and 33 (Table S11).

**Fig. 7:**
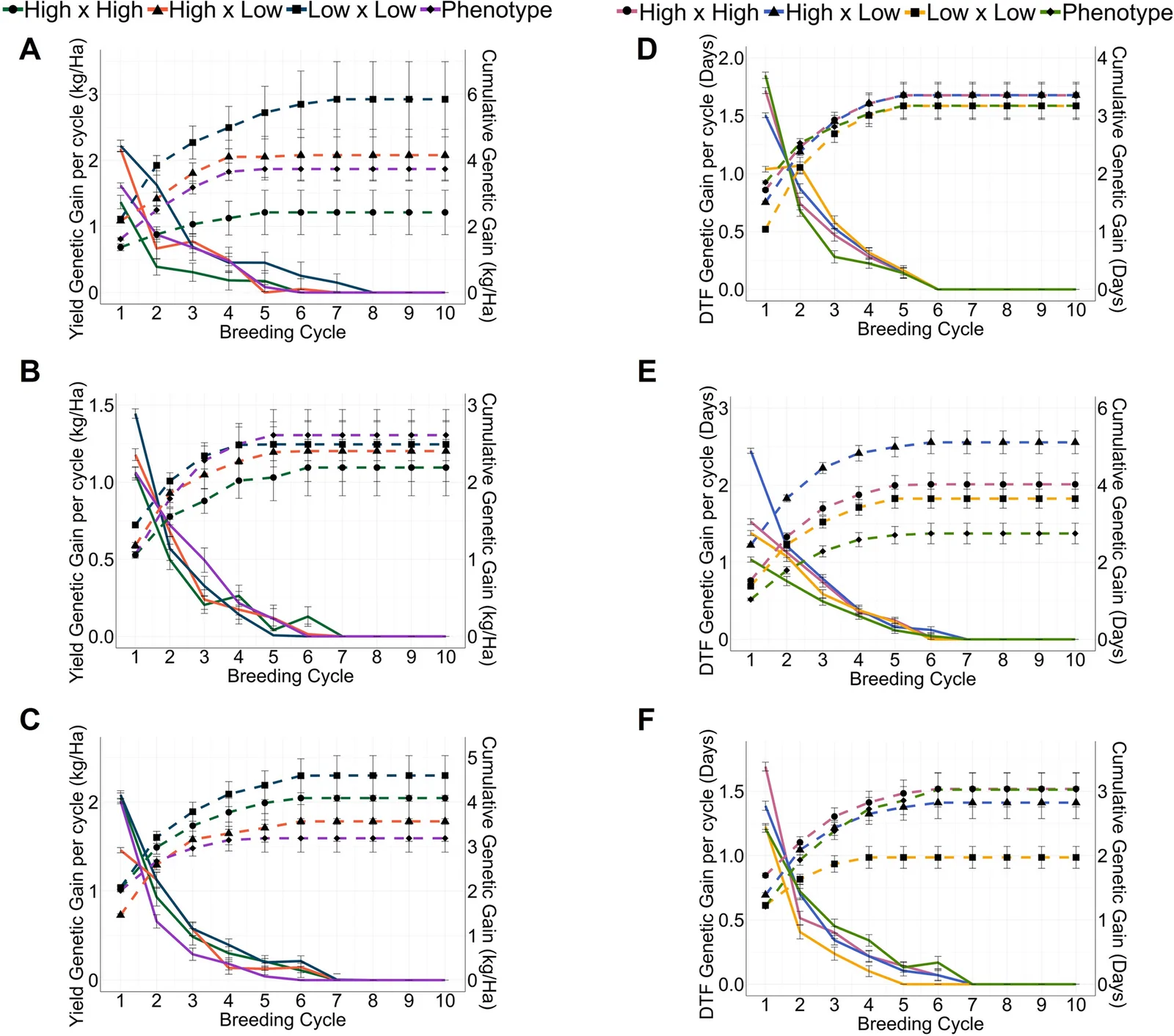
Simulation of genetic gain per cycle and cumulative genetic gain for yield (**A**-**C**) and days to flowering (**D**-**F**) for the Vivero Equipo Frijol (VEF; **A**, **D**), Black bean MAGIC (**B**, **E**), and Cooperative Dry Bean Nursery (CDBN; **C**, **F**) populations. High × High, High × Low, and Low × Low indicate the crossing schemes based on the number of highly deleterious mutations in each population. Phenotype is the phenotypic selection strategy based on BLUEs or BLUPs for top yield and low DTF

With the main H^2^ set at 0.25, the genetic gain for yield decreased every cycle in each population. In the first breeding cycle of the derived population from VEF, the genetic gain was 1.37 kg/Ha in the H × H scheme, 2.17 kg/Ha in the H × L scheme, 2.22 kg/Ha in the L × L, and 1.62 kg/Ha in the phenotypic selection. In the second cycle, L × L showed a higher genetic gain (1.63 kg/Ha) compared to the other three strategies (0.39–0.68 kg/Ha). The increase in genetic gain was close to zero after the fifth cycle for H × L, H × H, and phenotypic selection crossing schemes, whereas small increases were observed for the L × L strategy until the seventh cycle (Fig. 7A). In the cumulative genetic gain and H^2^ = 0.25, higher increases were observed for the crosses made from parents with a low number of *HDelM* (L × L) with a final gain of 5.85 kg/Ha (0.54%) followed by the H × L crosses with 4.16 kg/Ha (0.39%), the phenotypic selection crosses with 3.74 kg/Ha (0.35%), and finally the H × H crosses with 2.43 kg/Ha (0.23%; Table 1). At lower (H^2^ = 0.15; Fig. S15A) and higher (H^2^ = 0.35; Fig. S15B) heritability values the crossing scheme based on phenotypic selection and a higher genetic gain (3.22 kg/Ha and 3.54 kg/Ha, respectively), compared to the L × L scheme (2.33 kg/Ha and 3.46 kg/Ha, respectively; Table 1).

**Table 1:** Cumulative genetic gain after 10 breeding cycles in each population according to the crossing scheme and varying set broad-sense heritability (H^2^). Heritability with * is the main value used in the study for comparisons. Underlined values are either the highest (yield) or lowest (DTF) genetic gain within each population and tested H^2^. Each value has its confidence interval

*Yield (Kg/Ha)*									
Crossing scheme	VEF H^2^			MAGIC H^2^			CDBN H^2^		
	0.15	0.25*	0.35	0.15	0.25*	0.35	0.15	0.25*	0.35
H × H	2.46 ± 0.39	2.43 ± 0.67	2.72 ± 0.27	2.76 ± 0.41	2.18 ± 0.36	3.55 ± 0.26	1.87 ± 0.23	4.09 ± 0.46	4.30 ± 0.43
H × L	2.42 ± 0.42	4.16 ± 0.78	3.45 ± 0.26	3.25 ± 0.51	2.40 ± 0.39	3.61 ± 0.32	3.89 ± 0.60	3.57 ± 0.45	5.16 ± 0.41
L × L	2.33 ± 0.43	5.85 ± 1.14	3.46 ± 0.27	4.21 ± 0.55	2.49 ± 0.29	2.74 ± 0.33	2.36 ± 0.37	4.59 ± 0.45	3.76 ± 0.31
Phenotype	3.22 ± 0.40	3.73 ± 0.33	3.54 ± 0.26	3.43 ± 0.52	2.61 ± 0.33	3.83 ± 0.27	2.09 ± 0.51	3.19 ± 0.31	3.21 ± 0.32

*Flowering (days to flowering)*									
Crossing scheme	VEF H^2^			MAGIC H^2^			CDBN H^2^		
	0.35	0.45*	0.55	0.35	0.45*	0.55	0.35	0.45*	0.55
H × H	2.38 ± 0.26	3.35 ± 0.22	3.22 ± 0.17	3.04 ± 0.27	4.01 ± 0.29	4.98 ± 0.26	2.91 ± 0.20	3.04 ± 0.24	4.14 ± 0.27
H × L	3.20 ± 0.28	3.35 ± 0.20	3.67 ± 0.21	3.12 ± 0.33	5.11 ± 0.29	5.01 ± 0.23	3.87 ± 0.34	2.82 ± 0.25	4.18 ± 0.27
L × L	4.03 ± 0.34	3.17 ± 0.23	4.40 ± 0.21	3.22 ± 0.26	3.65 ± 0.24	5.56 ± 0.26	1.06 ± 0.18	1.97 ± 0.17	3.78 ± 0.25
Phenotype	3.24 ± 0.28	3.17 ± 0.21	2.11 ± 0.19	3.58 ± 0.34	2.75 ± 0.27	3.22 ± 0.26	3.42 ± 0.31	3.02 ± 0.27	4.37 ± 0.24

Similar trends in the reduction of genetic gain per cycle and the increase of cumulative genetic gain were observed for the three selection strategies based on DelMut in the MAGIC and CDBN populations for yield with H^2^ = 0.25. In the MAGIC population, lower genetic gains were predicted (Fig. 7B). In the first cycle, the yield increase for the L × L scheme was 1.44 kg/Ha, 1.17 kg/Ha for H × L, 1.06 kg/Ha for the phenotypic selection, and 1.05 kg/Ha in H × H. By the tenth cycle, the cumulative genetic gain was lower in H × H (2.18 kg/Ha; 0.11%) compared to L × L (2.49 kg/Ha; 0.13%) and H × L (2.40 kg/Ha; 0.13%). Interestingly, for the MAGIC population, the selection based on phenotype had a slightly higher cumulative genetic gain after the fifth cycle, with a final gain of 2.61 kg/Ha (0.14%), close to L × L (2.49 kg/Ha) and H × L (2.40 kg/Ha; Table 1). Similar results were observed with higher heritability (H^2^ = 0.35; Table 1). However, when the heritability was lower (H^2^ = 0.15), the L × L scheme had a higher cumulative genetic gain (4.21 kg/Ha; Table 1).

In the simulation from the CDBN genotypes with the main H^2^ (0.25), the genetic gain in the first cycle was around 2.06 kg/Ha for the L × L and the H × H crossing schemes, followed by the phenotypic selection with 2.01 kg/Ha, and the H × L with 1.46 kg/Ha. The final cumulative genetic gain across crossing strategies was lower than VEF (but higher than in MAGIC). As in VEF, the cumulative genetic gain was highest in the L × L scheme, reaching 4.59 kg/Ha (0.15%). The H × H derived crosses had a final genetic gain of 4.09 kg/Ha (0.13%), followed by the H × L scheme with 3.57 kg/Ha (0.12%). In CDBN, the phenotypic selection had the lowest cumulative genetic gain (3.19 kg/Ha (0.11%); Fig. 7C). In the simulations with a lower (0.15) or higher (0.35) heritability, the H × L scheme has consistently higher cumulative genetic gains (3.89 and 5.16 kg/Ha, respectively) compared to the other crossing strategies (Table 1).

Low genetic gains for flowering were observed in the simulated cycles from the VEF and CDBN populations with the main heritability (0.45). In the VEF simulations, phenotypic selection (1.85 days), H × H (1.72 days) and H × L (1.5 days) had higher genetic gains in the first cycle compared to L × L (1.04 days). By the sixth cycle, all schemes no longer showed a genetic gain per cycle (Fig. 7D). Final cumulative genetic gains were similar for H × H and H × L (3.35 days; 8.5%) and slightly lower for L × L and phenotypic selection (3.17 days; 8.0%). When a lower H^2^ (0.35) was considered, the H × H scheme had the lowest increase in DTF (2.38 days), whereas with a higher H^2^ (0.55), phenotypic selection had the lowest value (2.11 days; Table 1).

For the MAGIC and CDBN simulations when H^2^ was set at 0.45, clearer differences among crossing schemes were observed. In MAGIC, higher overall genetic gains for flowering were observed compared to VEF and CDBN. In the first cycle, the H × L crosses gained 2.45 days, H × H 1.53 days, L × L 1.38 days, and phenotypic selection 1.04 days. This trend between crossing schemes was maintained in the cumulative genetic gain, with a final increase of 5.11 days (10.7%) in H × L, 4.01 days (8.5%) in H × H, 3.65 days (7.7%) in L × L, and 2.75 days (5.8%) in phenotypic selection. A similar trend was observed when the H^2^ was set at 0.55, with lower increases when phenotypic selection was used (3.22 days; Table 1). Interestingly, with a H^2^ of 0.35, the H × H scheme had the lowest simulated increase (3.04 days) in DTF across schemes and H^2^ (Table 1).

For the CDBN simulation with H^2^ = 0.45, the L × L strategy had lower genetic gains in DTF compared to the other strategies and reached zero at the fourth cycle. The H × H, H × L, and phenotypic selection schemes registered gains until the sixth cycle (Fig. 7F). The final cumulative gain in DTF was higher in H × H (3.04 days; 5.1%), phenotypic selection (3.02 days; 5.1%), and H × L (2.82 days; 4.8%) than in the L × L cycles (1.97 days; 3.3%) when H^2^ was set at 0.45. Independently of the H^2^ value, the L × L scheme had the lowest gain in DTF with a minimum of 1.06 days when was H^2^ 0.35 (Table 1).

## Discussion

### Deleteriousness scores reveal a predominance of mild, near-neutral effects with rare large-impact variants

We investigated how the prediction of DelMut can potentially inform selection decisions and accelerate breeding programs. We evaluated three publicly available common bean breeding populations with diverse genetic and breeding histories. The adopted approach combines phylogenetic information with the likely impact of amino acid substitutions, providing a continuous classification of deleteriousness for variant prioritization (Cordoba-Novoa et al. 2025; Long et al. 2023; Ramstein and Buckler 2022). Based on this, the observed scores had an expected distribution in the three populations where most of the annotated SNPs have low values around 0.25. Mutations with a low score are likely to be tolerated, while mutations with a high score are predicted to be *highly* deleterious (Cordoba-Novoa et al. 2025; Long et al. 2023).

When considered individually, the effects of DelMut may seem inappreciable. It is the aggregated effect of multiple putatively DelMut with subtle individual effects that may have an impact on the overall plant fitness (Felsenstein 1974; Kono et al. 2019). Fitness refers to the reproductive success of an individual or population in certain environment (Orr 2009). While agronomic and fitness traits are not directly equivalent, yield and flowering can be indicatives of plant reproductive success. In common bean, yield depends on seed production, related to the relative representation of the plant in the next generation (reproductive success). Depending on the environment and agronomic context, flowering time can contribute to the plant development, establishment, and reproduction (Gaudinier and Blackman 2020). The distribution of the fitness effects of mutations is a research topic itself and explains the proportion of mutations that may be deleterious, neutral, or advantageous within populations (Eyre-Walker and Keightley 2007). Mutation accumulation experiments and other studies have shown that the effects of mildly DelMut generally follow a leptokurtic gamma distribution with a shape parameter (*α*) lower than one (Eyre-Walker et al. 2006; Keightley 1996). Here, we estimated the SNP effects on fitness-related traits based on the relationship between phenotypes and genetic markers using statistical models. While different from experimental approaches, this allowed us to compare the effects of allele changes (SNPs) predicted as deleterious with the effects of random SNPs (no deleterious) on two fitness-related traits, yield, and DTF. When analyzed as absolute values, the estimated effects for the scored SNPs (and above a certain threshold) had a highly leptokurtic gamma distribution in yield and flowering (Figs. 4 and 5). This indicates that most of the mutations are mildly deleterious, and a few of them have large (positive or negative) fitness effects in the long right tail of the distribution (Keightley 1996). These results are consistent with the distribution of the predicted scores (Fig. 3A-C) and the observed distributions within scored SNPs (Figs. S11 and S12). Additionally, Ohta and Kimura (1971) suggested that mutations are not equally deleterious but range from near-neutral to slightly deleterious to very deleterious alleles.

Leptokurtic distributions with low shape parameters are characterized by the clustering of values at the minimum (close to zero–low effect; Böndel et al. 2022; Piganeau and Eyre-Walker 2003). In the biological context of DelMut, large-effect mutations that can be lethal or highly detrimental to plant fitness are under strong purifying selection and eventually eliminated from the population. However, small-effect mutations can be tolerated and accumulated at a higher rate. If mildly DelMut had higher effects, they would probably be purged out as they may affect plant fitness. This may explain why scored SNPs in general and those with higher deleterious score values have estimated effects lower than random or low score SNPs. Mutations with higher scores are predicted to have more significant impacts on plant fitness compared to other variants and may be more informative for selection approaches.

Domestication and breeding reduce effective population sizes, increase inbreeding, and reduce the effectiveness of purifying selection, which leads to the accumulation of mildly deleterious mutations and a few *HDelM* (Grossen and Ramakrishnan 2024; Kono et al. 2016). This can be observed when analyzing the differences between the total genetic load in the subpopulations of CDBN. The races Durango and Mesoamerica belong to the primary Middle American gene pool, whereas race Nueva Granada belongs to the Andean gene pool. During common bean domestication and divergence, the Andean beans had a stronger bottleneck with smaller and relatively stable effective population sizes compared to Middle American beans for ~ 76,000 years after founding, resulting in less diversity (Mamidi et al. 2012; Schmutz et al. 2014). These differences in genetic background and domestication may result in a higher accumulation of mildly deleterious mutations in Andean beans. Additional research with high density genotypic datasets such as the one for CDBN, and a larger, more diverse population could elucidate additional patters of accumulation of DelMut in Andean beans.

Due to their low effect, slightly deleterious mutations have low selection coefficients (Hughes 2008). Under the nearly neutral theory, such mutations can behave as neutral and segregate at varying frequencies, potentially drifting to high frequency or fixation, when the effective population size is small (Ohta 1973). It is possible that deleterious mutations identified based on the predicted score act as neutral in the studied populations. This may explain the slight increase in the density of SNPs with intermediate MAF (0.1–0.4) in MAGIC and CDBN rather than very low (rare variants) or high frequencies (fixed variants) in the post filtering re-introduction.

### Incorporating predicted deleterious variants improves genomic prediction in a model- and trait-dependent manner

Factors such as population and model and variant selection affect the PA of GP models (Alemu et al. 2024; VanRaden et al. 2017), and strategies such as variant selection and differential marker weighing have been proposed to improve genomic selection efforts. We used two models that differ in how markers are treated. GBLUP assumes that all markers contribute equally to the total genetic variance, modeling breeding values through a genomic relationship matrix. Based on a Bayesian framework, BRR directly models marker effects, offering more flexibility, and has been applied to GP in common bean (de los Campos et al. 2013; Keller et al. 2020).

We consider how conservation and predicted deleteriousness can assist variant prioritization to optimize GP. In our results, differentially weighing SNPs based on their predicted deleteriousness scores in GBLUP did not improve PA for yield. However, the partition and independent modeling of scored and unscored SNPs as separate random effects in the BRR model, improved the PA of BRR for yield in the VEF and CDBN populations, with no clear effect on the MAGIC population (Fig. 6A). Interestingly, a higher gain in PA with multi-kernel BRR was observed in the VEF population which is a set of elite varieties, but with lower yields compared to MAGIC and CDBN as described above. VanRaden et al. (2017) and Xavier (2019) highlight the importance of variant selection, pruning, and filtering to enhance genomic prediction. Causal variants and markers closer to them or in strong linkage are expected to explain higher proportions of variance compared to random markers (Meuwissen et al. 2024). Very conservative filtering methods may result in losses in PA, while an excessive number of markers increase computational burdens. This can explain how BRR benefited from a different modeling of SNPs, whereas weighing in GBLUP decreased PA. Although weighted GP approaches can improve prediction models with varying success (Fang et al. 2017; Long et al. 2023; Meuwissen et al. 2024; Zhang et al. 2010, 2024), improvements to GBLUP can vary depending on the applied weights, trait architecture, population size, and characteristics potentially explaining the observed results (Lourenco et al. 2017).

Yield is a highly complex trait controlled by multiple small-effect genes and their interactions. One characteristic of the scored SNPs from the RF model is that they are in coding regions (Cordoba-Novoa et al. 2025; Ramstein and Buckler 2022). In the BRR model, it is possible that prioritizing putatively DelMut as independent random effects could help the model capture a substantial and biologically relevant *cumulative* effect (de los Campos et al. 2013; Goddard et al. 2010). Another plausible explanation for the modest improvements on PA of BRR is that prioritized SNPs (scored) can be in a strong linkage disequilibrium (LD) with unknown causal variants, contributing to a better capture of the genetic variation and prediction (Vilhjálmsson and Nordborg 2012). Kono et al. (2019) showed in barley that top progeny selected based on an RR-BLUP model had fewer DelMut relative to other lines. This suggests that DelMut affect the observed phenotypes and GP models indirectly account for putatively DelMut. In this regard, in other self-pollinating species such as soybean, elite lines have been shown to have a lower number of DelMut (Kim et al. 2021). Negative correlations between DelMut (and genetic loads) and yield have also been reported for common bean (Cordoba-Novoa et al. 2025), maize (Yang et al. 2017), and potato hybrid populations (Wu et al. 2023).

The VEF population was previously evaluated in multiple environments and traits using a genomic prediction framework. The PAs we obtained for yield and flowering with either GBLUP or BRR follow the previous study (Keller et al. 2020). Keller et al. (2020) found that the inclusion of significant QTL signals as fixed effects did not improve the PA of the model. Instead of accounting for fixed effects, our approach directly modifies how SNPs are accounted for in the model based on deleterious score, showing consistent PA improvements with the BRR model in VEF and other populations for yield.

Predictions for days to flowering (DTF) did not benefit considerably from incorporating DelMut information (either weights or multi-kernel partition) into the model. On the contrary, for the MAGIC and CDBN populations, the weighted GBLUP had a lower PA. Flowering time is a less complex trait than yield, with a larger proportion of phenotypic variance attributed to genetic factors (higher broad-sense heritability). As a less complex trait, flowering time is controlled by fewer, bigger-effect genes compared to yield. A higher number of small-effect genes involved in the multiple subprocesses affecting yield also lead to a higher accumulation of DelMut affecting the trait (more genes accumulate more DelMut related to the trait). This may explain why the prediction of yield is more responsive to the inclusion of DelMut information in the GP models.

In contrast to yield, significant QTL and genes with high effect have been identified through linkage mapping and GWAS for DTF (Ates et al. 2018; Nascimento et al. 2018; Raggi et al. 2019). Given that these QTLs account for a significant percentage of the phenotypic variation, additive genetic effects may already be effectively captured in the model, with no apparent advantages from SNPs with minor effects, such as the scored ones. When examining the effects of marker selection for GP in radiata pine (*Pinus radiata*) and shining gum (*Eucalyptus nitens*), Klápště et al. (2020) also noted that low heritability traits benefit more from model refinement. In simulation studies, Morgante et al. (2018) demonstrated that the prediction of traits governed by a greater number of QTLs (more complex) is more susceptible to parameter tuning compared to traits of lower complexity. This may explain why a multi-kernel approach with BRR improved the PA for yield, but had no clear effect on DTF. Additional research regarding weighing strategies and models that benefit the most from DelMut information is still needed. Machine learning and artificial intelligence may assist the analysis of DelMut in GP applications.

### Selection against highly deleterious mutations improves simulated genetic gain

Stochastic simulations support the optimization of breeding programs under varying parameters. Mating system designs influence the efficiency of breeding cycles and the rate of genetic gain over time. Here, we established a 1% deleterious score threshold to define *highly* deleterious mutations and assist the selection of promising material. More notably, this is an extension of the traditional characterization of DelMut with an *in silico* practical implementation. This threshold was defined to facilitate the characterization of DelMut across populations with the confidence of capturing highly relevant mutations. In this regard, when evaluating a model based on an evolutionary approach like the one we implemented, Zhai et al. (2025) found that many known phenotype-impacting and potentially deleterious mutations are found in the top 1% characterized mutations. As one of the first reports of the potential and practical use of the identification of deleterious mutations, we expect to define a baseline that serves to identify future improvements. Additional research in parameter tuning or thresholds can help optimize selection efforts and maximize genetic gains in the longer term.

In our simulated closed system, the selection of parents based on the number of *HDelM* influenced the expected genetic gain over time. For yield (H^2^ = 0.25), higher per cycle and cumulative genetic gains were consistently observed in all the populations for mating schemes where the initial parents had a low number of *HDelM* (L × L), except for MAGIC where the phenotypic selection had a slightly higher cumulative genetic gain. Based on the simulations in VEF and CDBN, while efficient in reaching acceptable genetic gains, phenotypic selection may be further improved by considering the number of DelMut in promising parents. Empirical validation and application of the proposed framework are necessary to corroborate this hypothesis.

Since the genetic load of plants does not have drastic phenotypic effects and DelMut are not routinely considered in GP schemes, our results support the potential of predicting and considering DelMut in breeding populations to inform selection decisions, particularly in the choice of parents. In animal breeding, Sonesson et al. (2003) and Raoul et al. (2018) stress the value of selecting against carriers and designing mating systems based on the incorporation or exclusion of these individuals. Carriers are individuals known to carry recessive mutations associated with diseases that could express later in the breeding process. Johnsson et al. (2019) simulated how the selection against carriers and genome editing of DelMut increase animal average fitness. They observed that the most effective strategy depends on the DelMut level of dominance (codominant or recessive). Selection against carriers is more effective for recessive mutations, whereas genome editing has the potential for both. Following our results, similar applications could be incorporated into plant breeding.

Different gene action models have been proposed for DelMut, including additive, incomplete dominance, and codominance (Robinson et al. 2023; Sun et al. 2023). The observed improvements in GP using BRR and the final simulated genetic gain in each crossing scheme may be attributed to the independent small additive effects predicted for DelMut. However, in other species such as maize, incomplete dominance of DelMut can also contribute to trait variation and heterosis (Yang et al. 2017). Additional research on other gene action effects of DelMut may open new avenues on how effects are modeled and then incorporated in breeding programs.

In our cumulative genetic gain of DTF (H^2^ = 0.45), breeding initiated with parents with a low number of *HDelM* (L × L) or phenotypic selection in MAGIC had a lower gain. Short flowering time has been positively correlated with yield in previous studies (Kamfwa et al. 2015; Moghaddam et al. 2016). It is possible that when selecting parents against a high number of *HDelM*, unknown mutations in genes involved in flowering are indirectly excluded, particularly in the first breeding cycle. For instance, the repair of a domestication DelMut in a floral regulator induced early fruit yield and more compact plants in tomato (Glaus et al. 2025). After the first cycle, parents are selected based on simulated phenotypes (top ones), which, based on the relationship between yield and flowering, can influence a lower flowering time in the cumulative genetic gain. Based on our results, the selection and design of mating (crossing) schemes between parents with a low number of predicted DelMut (L × L) has the potential to increase the genetic gain in yield and reduce flowering time. However, it is important to highlight that results may vary depending on the population characteristics.

Heritability estimations are experimental and population dependent. However, common H^2^ values facilitated some comparisons across populations. At the main and lower H^2^ values for yield and DTF, selection strategies and mating schemes based on the number of *HDelM* showed consistently higher genetic gains in the populations, except for yield in the VEF. When heritability was increased, phenotypic selection showed a better performance in increasing the cumulative genetic gain for yield and DTF in VEF and MAGIC populations. However, for the CDBN population, mating schemes with individuals selected to have a low number of *HDelM* had higher genetic gains. These results could be attributed to how traits with low heritability and polygenic architecture may benefit more from genomic-based approaches (Vieira et al. 2025) compared to traits with high heritability where phenotypic-based approaches can be sufficient. It is possible that in traits with low heritability the selection of parents with fewer *HDelM* supports the exclusion of multiple alleles with varying levels of deleteriousness that are not easy to observe in the phenotype. As a population and trait-dependent approach, our results provide a primary baseline for future experiments aimed at evaluating the effect of varying multiple factors in simulation or empirical selection experiments.

Different responses to prediction and simulation were observed between populations. As mentioned above, characteristics proper to each population limit direct comparisons and restrict results within each one. In a practical setting, each breeding program can evaluate the presence and inclusion of DelMut in the context of the already-implemented genotyping and selection approaches. Our results show consistent patterns of how the information on DelMut can benefit both GP and the design of crossing blocks. The MAGIC population had a lower PA and simulated genetic gains in yield compared to VEF and CDBN. Multiparent populations are designed to accumulate a greater number of recombination events, and the progeny lines are mosaics with contributions of all parents (Gardner et al. 2016; Wang et al. 2022). These advantages make MAGIC populations particularly useful for gene mapping with a higher resolution, trait introgression, and marker-assisted selection. As more recombination events are accumulated, the linkage between alleles can be broken and allelic diversity increased. While this is desirable for eliminating DelMut in LD with beneficial alleles, this may also break the linkage between two or more positive alleles in coupling phase. In fact, Tourrette et al. (2019) and Taagen et al. (2022) showed that increased recombination, either in pericentromeric or chromosome-wide regions, can be detrimental for genetic gain and GP accuracies when it breaks up QTL in coupling phase. An increased recombination is particularly beneficial when positive QTL are in repulsion phase or targeted recombination approaches are adopted (Ru and Bernardo 2019). These may partly explain why the MAGIC population had lower PAs and simulated genetic gains, especially for yield where more QTL are expected to contribute to the trait variation (more complex). Additionally, Keller et al. (2020) observed lower PA in a different MAGIC population compared to the same VEF population used here.

As proof of concept, the adopted approach has some limitations. In breeding programs, after each breeding cycle, new parents are integrated into the pipeline to preserve genetic diversity and continue increasing the genetic gain linearly. In our simulations, the genetic gain per cycle decreases after each cycle as alleles are fixed and the inbreeding increases. For the same reason, the cumulative genetic gain reaches a plateau value with no further increases. Here, we demonstrated in silico how the selection against DelMut has the potential to make breeding programs more efficient even over phenotypic selection in some populations. Once empirically validated, these strategies can complement and assist breeders’ work. Future simulations and empirical studies should evaluate simulations with the re-introduction of new germplasm similarly selected based on the number of DelMut. As a multifactor process, breeding programs are complex. Simulations that include changes in other parameters and evaluate the expected response of parents selected based on DelMut would also be valuable.

## Conclusions

We evaluated whether predicting deleterious mutations in common bean breeding populations can inform genomic prediction (GP) and increase the rate of genetic gain for yield and flowering time. The absolute value of the estimated effects of variants with predicted scores and with higher score values had a highly leptokurtic gamma distribution, different from that of random and lower-scored markers. Incorporating deleterious scores as weights in the GRM of a GBLUP model reduced PA, but a multi-kernel approach in BRR where scored markers are independently modeled improved yield prediction by 6.0–18.6% relative. For DTF, marginal improvements were observed (0.3–2.27% relative) depending on the model and population. The selection of parents with fewer *highly* DelMut (HDelM) has the potential of increasing the genetic gain in yield and flowering depending on the population. For instance, in the VEF population, crosses between genotypes with seven to eight *HDelM* had a higher cumulative genetic gain for yield (5.85 kg/Ha) compared to crosses with 12–13 *HDelM* (2.43 kg/Ha) and phenotypically selected (3.74 kg/Ha). For flowering time, crossing schemes with fewer *HDelM* also showed more favorable DTF changes in VEF and CDBN. These findings demonstrate the potential of DelMut information to support breeding program optimization through GP and simulation-based approaches.

## Supplementary Information

Below is the link to the electronic supplementary material.

Supplementary file1 (DOCX 5814 KB)

Supplementary file2 (XLSX 2900 KB)

## Data Availability

Code and materials used in this study can be found in the McGill University Pulse Breeding and Genetics GitHub page (https://github.com/McGillHaricots/peas-andlove).
