# Supplementary material for: Prioritization of deleterious mutations informs genomic prediction and increases the rate of gain in common bean (Phaseolus vulgaris L.), a simulation study
Source: Theor Appl Genet. 2026 Aug 3;139(8):220. doi: 10.1007/s00122-026-05329-z (PMC13433502; doi:10.1007/s00122-026-05329-z)
Supplement: Supplementary file 1 [file 122_2026_5329_MOESM1_ESM.docx]

**Supplementary Material**

**Figure S1.** Linkage Disequilibrium (LD) heatmaps for chromosomes 2 – 11 in Vivero Equipo Frijol (VEF) population.

**Figure S2.** Linkage Disequilibrium (LD) heatmaps for chromosomes 2 – 11 in black bean MAGIC population.

**Figure S3.** Linkage Disequilibrium (LD) heatmaps for chromosomes 2 – 11 in Cooperative Dry Bean Nursery (CDBN) population.

**Figure S4.** Distribution of the homozygous genetic load for the top 1% of the deleterious scores in Vivero Equipo Frijol (VEF; A), Black bean MAGIC population (B), and Cooperative Dry Bean Nursery (CDBN; C).

**Figure S5.** Distribution of the raw estimated effects of scored and unscored SNPs for yield in Vivero Equipo Frijol (VEF; A), Black bean MAGIC population (C), and Cooperative Dry Bean Nursery (CDBN; E). Cumulative probability for the distribution of the effects (B-F). D and p-value correspond to the Kolmogorov–Smirnov (KS) test for distributions. The SNP dataset is independent for each population.

**Figure S6.** Distribution of the raw estimated effects of scored and unscored SNPs for days to flowering (DTF) in Vivero Equipo Frijol (VEF; A), Black bean MAGIC population (C), and Cooperative Dry Bean Nursery (CDBN; E). Cumulative probability for the distribution of the effects (B, D, F). D and p-value correspond to the Kolmogorov–Smirnov (KS) test for distributions. The SNP dataset is independent for each population.

**Figure S7.** Distribution of the raw estimated effects of scored SNPs for yield according to the 30% of the distribution of deleterious scores threshold. Vivero Equipo Frijol (VEF; A, B), Black bean MAGIC population (C, D), and Cooperative Dry Bean Nursery (CDBN; E, F). Cumulative probability for the distribution of the effects (B, D, F). D and p-value correspond to the Kolmogorov–Smirnov (KS) test for distributions.

**Figure S8.** Distribution of the raw estimated effect of scored SNPs for days to flowering according to the 30% of the distribution of deleterious scores threshold. Vivero Equipo Frijol (VEF; A, B), Black bean MAGIC population (C, D), and Cooperative Dry Bean Nursery (CDBN; E, F). Cumulative probability for the distribution of the effects (B, D, F). D and p-value correspond to the Kolmogorov–Smirnov (KS) test for distributions.

**Figure S9.** Relationship between Minor Allele Frequency (MAF) and estimated effect on yield for SNPs with score (A, C, E) and with no score (B, D, F) in Vivero Equipo Frijol (VEF; A, B), Black bean MAGIC population (C, D), and Cooperative Dry Bean Nursery (CDBN; E, F).

**Figure S10.** Relationship between Minor Allele Frequency (MAF) and estimated effect on days to flowering for SNPs with score (A, C, E) and with no score (B, D, F) in Vivero Equipo Frijol (VEF; A, B), Black bean MAGIC population (C, D), and Cooperative Dry Bean Nursery (CDBN; E, F).

**Figure S11.** Distribution of the *absolute value* of the estimated effects of scored SNPs for yield according to the 30% of the distribution of deleterious scores threshold. Vivero Equipo Frijol (VEF; A, B), Black bean MAGIC population (C, D), and Cooperative Dry Bean Nursery (CDBN; E, F). Cumulative probability for the distribution of the effects (B, D, F). D and p-value correspond to the Kolmogorov–Smirnov (KS) test for distributions.

**Figure S12.** Distribution of the *absolute value* of the estimated effect of scored SNPs for days to flowering according to the 30% of the distribution of deleterious scores threshold. Vivero Equipo Frijol (VEF; A, B), Black bean MAGIC population (C, D), and Cooperative Dry Bean Nursery (CDBN; E, F). Cumulative probability for the distribution of the effects (B, D, F). D and p-value correspond to the Kolmogorov–Smirnov (KS) test for distributions.

**Figure S13.** Trace plots for the BRR models used for genomic prediction of yield in Vivero Equipo Frijol (VEF; A, B), Black bean MAGIC population (C, D), and Cooperative Dry Bean Nursery (CDBN; E, F) using a single kernel (A, C, E) or a multi-kernel approach (B, D, F).

**Figure S14.** Trace plots for the BRR models used for genomic prediction of days to flowering in Vivero Equipo Frijol (VEF; A, B), Black bean MAGIC population (C, D), and Cooperative Dry Bean Nursery (CDBN; E, F) using a single kernel (A, C, E) or a multi-kernel approach (B, D, F).

**Figure S15.** Linkage Disequilibrium (LD) heatmaps for chromosomes 1 – 11 in black bean MAGIC population after reduction based on LD (R^2^) (left side) and after reduction and re-introduction of scored SNPs (right side). For illustration purposes, the first 10 Mbp of each chromosome are shown.

**Figure S16.** Linkage Disequilibrium (LD) heatmaps for chromosomes 1 – 11 in Cooperative Dry Bean Nursery (CDBN) population after reduction based on LD (R^2^) (left side) and after reduction and re-introduction of scored SNPs (right side). For illustration purposes, the first 2 Mbp of each chromosome are shown.

**Figure S17.** Distribution of minor allele frequency (MAF) for genotypic datasets after filtering based on LD and after filtering and re-incorporation of scored SPNs in Black bean MAGIC population (A, B), and Cooperative Dry Bean Nursery (CDBN; C, D). Cumulative probability for the distribution of MAF (B, D). D and p-value correspond to the Kolmogorov–Smirnov (KS) test for distributions.
